# Plasma-Enhanced Atomic Layer Deposition of TiN Thin Films as an Effective Se Diffusion Barrier for CIGS Solar Cells

**DOI:** 10.3390/nano11020370

**Published:** 2021-02-02

**Authors:** Hyun-Jae Woo, Woo-Jae Lee, Eun-Kyong Koh, Seung Il Jang, Shinho Kim, Hyoungseok Moon, Se-Hun Kwon

**Affiliations:** 1 School of Materials Science and Engineering, Pusan National University, Busan 46241, Korea; present_w@naver.com (H.-J.W.); alivedoc@naver.com (W.-J.L.); tjqn1121@naver.com (E.-K.K.); shinho@pusan.ac.kr (S.K.); 2 Home Appliance & Air Solution Company R&D Center, LG Electronics, 170 Seongsanpaechong-Ro, Seongsan-Gu, Changwon-Si, Geyeongsangnam-Do 51533, Korea; s1jang@daum.net; 3 Advanced Energy Materials and Components R&D Group, Korea Institute of Industrial Technology (KITECH), Busan 33032, Korea

**Keywords:** CIGS solar cells, plasma-enhanced atomic layer deposition, TiN, Se diffusion barrier

## Abstract

Plasma-enhanced atomic layer deposition (PEALD) of TiN thin films were investigated as an effective Se diffusion barrier layer for Cu (In, Ga) Se_2_ (CIGS) solar cells. Before the deposition of TiN thin film on CIGS solar cells, a saturated growth rate of 0.67 Å/cycle was confirmed using tetrakis(dimethylamido)titanium (TDMAT) and N_2_ plasma at 200 °C. Then, a Mo (≈30 nm)/PEALD-TiN (≈5 nm)/Mo (≈600 nm) back contact stack was fabricated to investigate the effects of PEALD-TiN thin films on the Se diffusion. After the selenization process, it was revealed that ≈5 nm-thick TiN thin films can effectively block Se diffusion and that only the top Mo layer prepared on the TiN thin films reacted with Se to form a MoSe_2_ layer. Without the TiN diffusion barrier layer, however, Se continuously diffused along the grain boundaries of the entire Mo back contact electrode. Finally, the adoption of a TiN diffusion barrier layer improved the photovoltaic efficiency of the CIGS solar cell by approximately 10%.

## 1. Introduction

As the demand for electricity increases, the environmental concerns also increase mainly owing to greenhouse gas emissions. Electricity-generation systems are moving towards decarbonized technology. With the development of a variety of renewable energy sources, photovoltaic technologies that produce electricity have attracted the most attention owing to their reliable, potentially infinite, and clean sunlight source. Among the various types of solar cells, Cu (In, Ga) Se_2_ (CIGS) thin-film solar cells have a high efficiency exceeding 20%, thanks to their high absorption coefficient and tunable band-gap energy [1]. A CIGS thin film is typically prepared by a three-stage co-evaporation or two-step process. In the three-stage co-evaporation method, Cu, In, Ga, and Se are simultaneously evaporated, depositing their layer on the substrate under high vacuum and high temperature conditions. It is hard to synthesize CIGS layers on a large scale using this technique, as controlling the composition for each element is difficult; moreover, this is a relatively expensive process [2,3,4]. In contrast, the two-step process involves precursor deposition and selenization. First, the CIG layer is deposited by multi-source sputtering using Cu, In, and Ga metallic precursors. Then, selenization is carried out using H_2_Se or Se vapor gas [5]. This two-step process has been recently preferred because of its low costs and large-area approach that can be realized with a non-vacuum atmospheric process, resulting in a relatively uniform composition of the CIGS layer.

As a back contact electrode of the CIGS solar cell, Mo is typically used owing to its high electrical conductivity, similar thermal expansion coefficient to that of a glass substrate, and suitable adhesion with CIGS. During the selenization process, the Mo back contact electrode reacts with Se to form the MoSe_2_ layer. The formed MoSe_2_ layer can facilitate ohmic contact and can promote adhesion between the CIGS layer and Mo electrodes, enhancing the CIGS performance in terms of efficiency [6]. However, uncontrolled and excessive Se diffusion from the CIGS layer to the Mo back contact electrode can destroy the optimal composition of the CIGS photo-absorbing layer and can reduce adhesion between the CIGS/MoSe_2_/Mo layers [7]. To maintain an appropriate thickness of the MoSe_2_ layer, many studies have adjusted the selenization and sputtering conditions. However, in these methods, controlling the process parameters and obtaining adequate reproductivity for large-scale production are fairly difficult and complex [8,9]. To minimize the adverse effects of cell performance, a diffusion barrier can be introduced, which effectively prevents excessive Se diffusion through a relatively simple process. As a material of the diffusion barrier that is chemically stable and has excellent barrier characteristics, transition metals and their nitrides have been reported for applications in highly integrated devices [10]. In particular, TiN is considered one of the most prominent materials for diffusion barriers owing to its high thermal stability, high barrier characteristics, and relatively low price. Furthermore, many researchers have reported that TiN thin films play a very effective role as barrier layers in copper wiring [11]. Among the deposition methods for preparing TiN thin films, plasma enhanced-atomic layer deposition (PEALD) is considered a highly promising technique to obtain high-quality TiN thin films at relatively low temperatures using the high reactivity of plasma. Also, PEALD is capable of offering precise thickness control by the self-limiting reaction mechanism of atomic layer deposition (ALD), and it offers excellent step coverage and uniformity [12]. Due to its excellent characteristics, the ALD process is necessary and regarded as a cost-efficient technique in large scale and high-volume manufacturing of solar cell [13,14].

In this study, therefore, an ultrathin TIN diffusion barrier was introduced into a Mo back contact electrode to control Se diffusion at the interface between the CIGS absorption layer and Mo electrode. A TiN layer was deposited by PEALD with tetrakis(dimethylamido)titanium (TDMAT) and N_2_ plasma. The effects of the TiN diffusion barrier were systematically identified by analyzing the chemical profiling, microstructure and energy conversion efficiency of CIGS solar cells by comparing the Mo single layer and Mo/ TiN/Mo layer.

## 2. Materials and Methods

TiN thin films were prepared at a deposition temperature of 200 °C via the PEALD method using TDMAT precursors and N_2_ plasma. To optimize the PEALD-TiN process, a 250 nm-thick SiO_2_/Si substrate was used. For a sufficient supply of TDMAT, TDMAT was contained in a bubbler and maintained at a temperature of 40 °C. One deposition cycle of TiN consisted of a TDMAT precursor injection with 25 sccm Ar carrier gas for 1 s, a purge pulse with 50 sccm Ar for 10 s, a pulse for the N_2_ plasma exposure with 100 sccm N_2_ gas for 10 s, and another 50 sccm Ar purge pulse for 10 s. During the PEALD-TiN processes, Ar gas was consistently supplied to the chamber at a flow rate of 50 sccm, and the chamber pressure was maintained at 3 Torr. During the plasma pulse, radiofrequency (RF) plasma was used at an electrical power of 300 W.

The film thickness was analyzed via field-emission scanning electron microscopy (FE-SEM, Hitachi S-4800). High-resolution transmission electron microscopy (HR-TEM) and energy-dispersive X-ray spectroscopy (EDS) were used to analyze the microstructure and elemental mapping (JEOL, JEM-2100F HR). The crystal structure was investigated via X-ray diffraction (XRD, Rigaku Ultima IV) with 1.54 Å Cu-K_α_ radiation. Chemical analysis and depth profiling were conducted via secondary ion mass spectrometry (SIMS, CAMECA IMS-7F) with a Cs^+^ primary beam at 10 keV with a current of 20 nA and ion beam raster size of 200 μm × 200 μm.

To examine the photovoltaic properties of the CIGS solar cells, two types of back contact stack were prepared, i.e., Mo (≈30 nm)/TiN (≈5 nm)/Mo (≈600 nm)/soda lime glass and Mo (≈630 nm)/soda lime glass, where Mo films were prepared using DC-sputtering method. Then, a CIGS solar cell stack was fabricated as follows. First, CIGS (1500 nm) thin films were deposited on two back contact stacks by a conventional two-step CIGS process. After the chemical bath deposition (CBD) of CdS (50 nm) films on the CIGS, the transparent conducting thin film of Al-doped ZnO (500 nm) was deposited by RF sputtering. An electrode pattern was finally formed using 120 nm-thick evaporated Al with an effective area of 0.43 cm^2^. The current–voltage (J–V) characteristics of the devices during illumination were measured using a Keithley 2400 source meter unit and solar simulator under the AM 1.5 standard illumination. 

## 3. Results and Discussion

**Figure 1:**
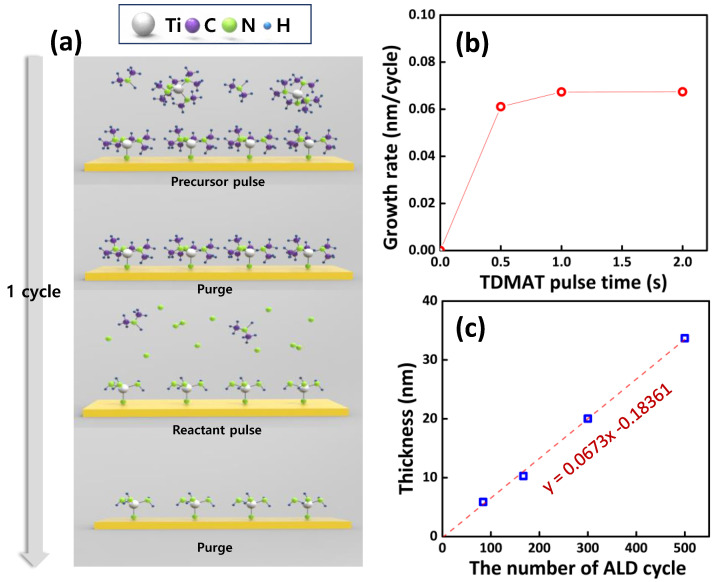
(**a**) Schematic diagram of one cycle of the plasma-enhanced atomic layer deposition (PEALD)-TiN process using tetrakis(dimethylamido)titanium (TDMAT) and N_2_ plasma. (**b**) Growth rate as a function of TDMAT precursor pulse time. (**c**) TiN thin-film thickness depending on the number of atomic layer deposition (ALD) cycles.

Before applying TiN thin films as a Se diffusion barrier layer, the self-limiting growth of the PEALD-TiN thin films was studied. Figure 1a presents a schematic diagram of one PEALD cycle for TiN thin films. First, the TDMAT precursors were injected into the chamber to be chemisorbed on the active sites of the SiO_2_ surface. During a purge step, excessive precursors and by-products were pumped out of the chamber. Then, the N_2_ plasma reactant was introduced. During the plasma pulse, reactive species can break the ligand of the chemisorbed TDMAT, leading to Ti–N bonding. In another purge step, by-products were also removed by Ar purge gas, finally synthesizing the monolayer of the TiN thin films. This cycle of four sequential steps was repeated to obtain the desired TiN thickness. Based on this fundamental process, the TiN growth rate was investigated as a function of the TDMAT precursor pulse time to confirm the ALD reaction mechanism, as illustrated in Figure 1b. Other sequences in a single cycle, except for the TDMAT precursor pulse time, were fixed to a 10 s purge, N_2_ plasma for 10 s, and another 10 s purge. By increasing the TDMAT pulse time from 0.5 s to 1 s, the growth rate increased and then saturated above 1 s. This behavior indicates the self-limiting reaction mechanism, a characteristic of ALD [15]. Figure 1c details the TiN thicknesses depending on the number of ALD cycles. The thicknesses increased linearly with an increase in cycles, which is a typical ALD characteristic; a growth rate of 0.67 Å/cycle was obtained. In addition, the extrapolated line approaches the origin, indicating the rapid nucleation of TiN deposition by PEALD. Based on this, it is confirmed that the TiN thin films were successfully deposited and optimized by the PEALD system using TDMAT and N_2_ plasma.

**Figure 2:**
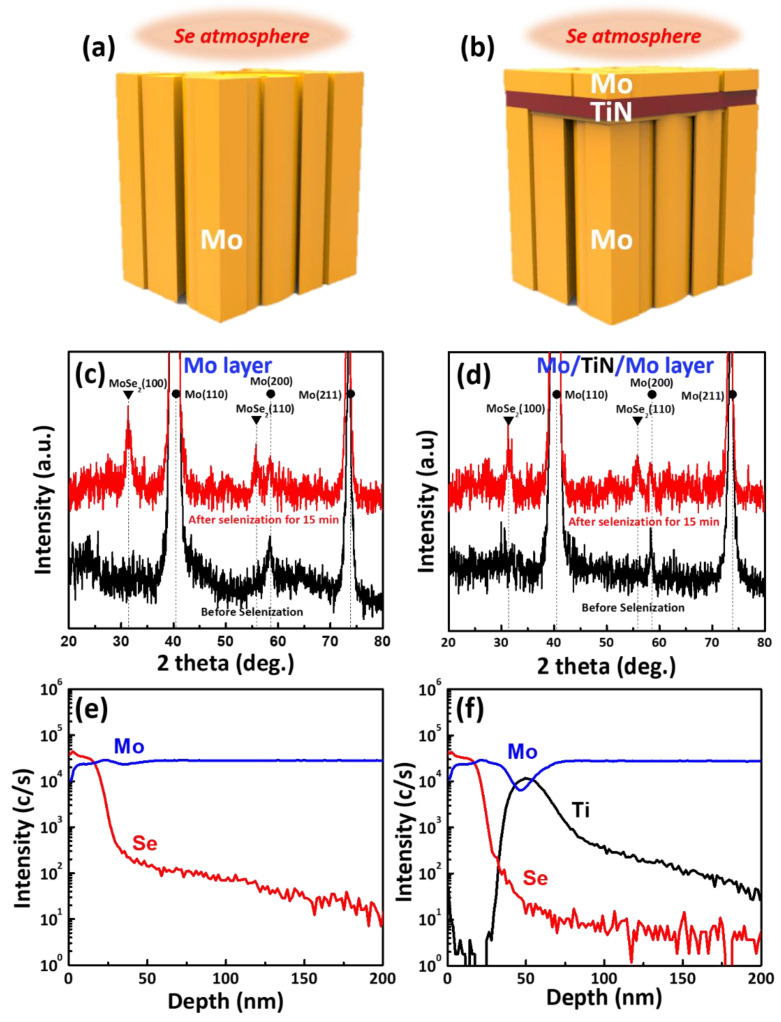
Schematic diagram of (**a**) a pure Mo layer and (**b**) a Mo/TiN/Mo multilayer, X-ray diffraction (XRD) analysis of (**c**) pure Mo layer and (**d**) Mo/TiN/Mo multilayer before and after selenization, and secondary ion mass spectrometry (SIMS) depth profiling of (**e**) a pure Mo layer and (**f**) a Mo/TiN/Mo multilayer after selenization.

To analyze the effects of the TiN layer as a Se diffusion barrier in the CIGS solar cell, about a 5 nm-thick TiN film was first deposited on the Mo back contact electrode; then, Mo was deposited again on the prepared TiN/Mo back contact electrode, forming the Mo/TiN/Mo structure. Next, selenization was performed for the Mo single electrode (Figure 2a) and Mo/TiN/Mo structure (Figure 2b) at 500 °C for 15 min under a Se atmosphere for comparison. Figure 2c,d present the XRD spectra of the Mo single and Mo/TiN/Mo back contact stacks after selenization, indicating MoSe_2_ (100) and (110) peaks with an HCP structure. This indicates that Se diffused into the Mo back contact electrode, forming the MoSe_2_ layer during the selenization process. However, the crystalline change or barrier effect by the TiN layer was hard to identify because of the similar XRD peak spectra. Therefore, the depth profiling of Mo, Se, and Ti elements was performed by SIMS analysis. As indicated by Figure 2e, the deeper the position, the lower the Se elements detected, indicating free diffusion into the Mo back contact electrode. For the Mo/TiN/Mo structure (Figure 2f), before approaching the TiN layer, the Se element in a Mo/TiN/Mo multilayer showed a similar decrease of pure Mo layer. After reaching the TiN layer, the intensity of Se in the Mo/TiN/Mo structure was lower than in the Mo single electrode; finally, the background signal of the Se was recorded at the Mo back contact electrode position [16]. This signal for Se inside the Mo single-layer structure was detected at a deeper position of the Mo layer, implying that the Se diffused deeper into the Mo electrode. This suggests that the TiN barrier layer can efficiently prevent undesired Se diffusion into the Mo single electrode.

**Figure 3:**
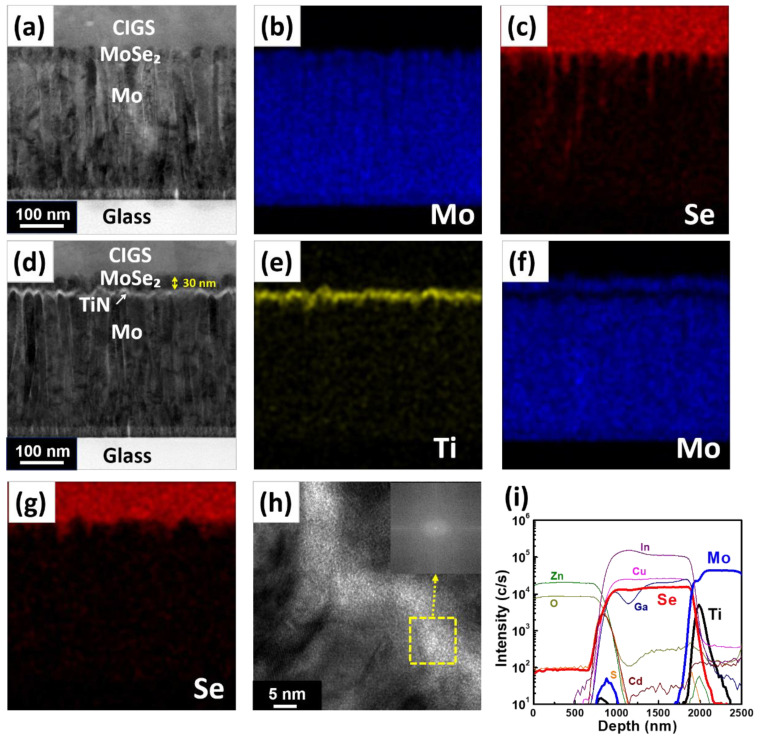
(**a**) Bright-field transmission electron microscopy (BF-TEM) image and energy-dispersive X-ray spectroscopy (EDS) elemental mapping image of (**b**) Mo and (**c**) Se for the Cu (In, Ga) Se_2_ (CIGS) solar cell without a TiN diffusion barrier layer; (**d**) BF-TEM image and EDS elemental mapping image of (**e**) Ti, (**f**) Mo, and (**g**) Se for the CIGS solar cell with a TiN diffusion barrier layer; (**h**) an high-resolution transmission electron microscopy (HR-TEM) image with fast Fourier transform (FFT) (inset image) of the TiN diffusion barrier layer; and (**i**) SIMS depth profiling of the CIGS solar cell structure with a TiN diffusion barrier layer.

To further explore the role of the TiN diffusion barrier layer in the CIGS solar cell structures, CIGS cells were fabricated on both a Mo single electrode and Mo/TiN/Mo structure. Figure 3a presents the cross-sectional bright-field TEM images of the CIGS solar cell on a Mo single back contact electrode, exhibiting the columnar structure of the Mo back contact electrode. Figure 3b,c detail the EDS analysis of Mo and Se for the CIGS solar cell on the Mo single layer, respectively. Se diffuses along the grain boundary of the columnar grains into a deeper position of the Mo single back contact electrode, which corresponds to the SIMS data. In the case of the CIGS/Mo/TiN/Mo structure, as illustrated in Figure 3d, the TiN layer was successfully deposited between the MoSe_2_ and Mo structures. The deposited TiN thin films were uniformly formed along the underlying rough Mo morphology. Figure 3e,f confirm that, as the EDS peak of Ti elements was conformally detected, a uniform TiN barrier layer formed on top of the Mo back contact electrode. Figure 3g,h verify that Se did not diffuse into the Mo electrode, thanks to the excellent diffusion barrier of the TiN thin film; MoSe_2_ only formed above the PEALD-TiN layer. Figure 3h magnifies the TiN layer in the CIGS/Mo/TiN/Mo structure, clearly revealing the uniform TiN thickness of approximately ≈5 nm. Furthermore, TiN thin films exhibited amorphous structures, which were confirmed by fast Fourier transform (FFT) patterns at the yellow TiN portions. It is expected that amorphous TiN features excellent diffusion barrier properties because the grain boundary of the crystalline structure can normally provide fast diffusion paths [17,18]. Figure 3i details the SIMS depth profiling of the solar cell structure consisting of Al-doped ZnO/CdS/CIGS/Mo/TiN/Mo layers, indicating that the TiN barrier layer can efficiently block the Se element diffusion in the solar cell.

**Figure 4:**
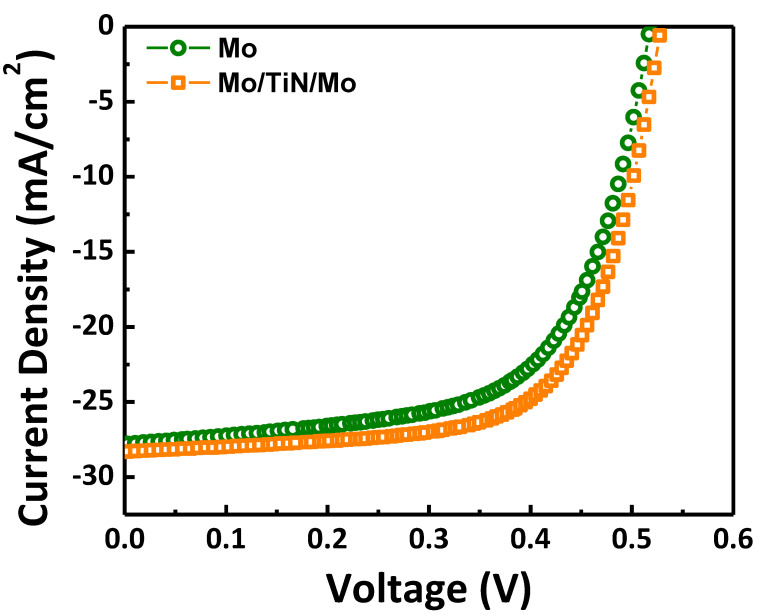
J–V curve of conventional and TiN buffer layer-inserted CIGS solar cells.

Finally, the performance of the CIGS solar cell fabricated on the Mo/TiN/Mo and Mo back contact electrode was each measured 12 times. Figure 4 shows a representative current density–voltage curve obtained from a sample with efficiencies most similar to the average efficiency value of 12 measurements. As detailed in Table 1, the sample with the TiN diffusion barrier layer exhibited higher V_oc_ (Open Circuit Voltage), FF (Fill Factor), J_sc_ (Short Circuit Current) Eff (Efficiency), and R_s_ (Series resistance) values than those of the CIGS solar cell without the TiN diffusion barrier layer. This result is probably due to the introduction of the TiN barrier layer, which efficiently diffuses Se in the CIGS layer into the Mo layer and synthesizes the appropriate MoSe_2_ layer [19]. According to the previous studies, MoSe_2_ layer is regarded as an important factor for the series resistance, J_sc_ and FF [20]. A properly controlled MoSe_2_ layer by introducing the TiN layer via PEALD, can contribute to lowering the series resistance due to the strong electrical contact between the CIGS absorber layer and Mo back contact, leading to the improved fill factor from the increased J_sc_. In addition, TiN with a higher reflectance than the Mo layer [21] may support the improvement in optical gain, leading to increased J_sc_ [22]. In the other words, CIGS solar cell on the Mo single layer without a TiN barrier layer can produce an excessively diffused layer of MoSe_2_ and, in turn, the Se deficiency of CIGS layer, resulting in lower cell efficiency due to a poor Mo ohmic contact and an insufficient back surface field [23,24]. It is found that the main enhancement in the TiN-introduced CIGS solar cell comes from the improved FF, which is attributed to its lower series resistance [25]. Overall, the PEALD-TiN layer inserted between the Mo layers can be used as an excellent diffusion barrier, enhancing the CIGS performance by controlling the MoSe_2_ layer and by preventing useless Se diffusion.

## 4. Conclusions

In this study, a TiN layer was introduced on the inside of the Mo layer to control the appropriate thickness of the MoSe_2_ layer, preventing Se from randomly diffusing into the Mo layer. TiN diffusion layer was successfully prepared by the PEALD process using a TDMAT precursor and N_2_ plasma. The performance of the TiN diffusion barrier layer was carefully analyzed after selenization process. SIMS analysis revealed that the diffusion of Se into the Mo back contact electrode was limited by inserting the TiN layer. To identify this behavior at the cell scale, a CIGS solar cell was fabricated onto the Mo single layer and Mo/TiN/Mo structure layer. For the Mo single layer, the TEM-EDS analysis revealed that Se diffused into a deeper position of the Mo layer along its columnar structure whereas the inserted TiN layer with an amorphous structure was uniformly deposited on the Mo electrode, diffusing Se effectively to the top of the TiN layer. Finally, the CIGS cell performance was observed, and the cell efficiency was enhanced by 10% when inserting the TiN barrier layer. Thus, it was concluded that the TiN diffusion layer via PEALD is a promising material to control Se diffusion.

## Figures and Tables

**Table 1:** Device characteristics of conventional and TiN diffusion barrier layer-inserted CIGS solar cells. Eff: efficiency, FF: fill factor, V_oc_: open circuit voltage, J_sc_: short circuit current, and R_s_: series resistance.

Back Contact Stack	Eff (%)	FF (%)	V_oc_ (V)	J_sc_ (A/cm^2^)	R_s_ (Ω)
Mo	9.055	62.86	0.5182	27.80	7.36
Mo/TiN/Mo	9.944	66.45	0.5286	28.31	7.03
